# The Future Is Bright for Women in Urologic Oncology: Trends over Two Decades

**DOI:** 10.3390/cancers18020310

**Published:** 2026-01-20

**Authors:** Gabrielle R. Yankelevich, Reid DeMass, Luis G. Medina, Tara Sweeney, Robert L. Grubb, Stephen J. Savage, Matvey Tsivian

**Affiliations:** 1 Department of Urology, Medical University of South Carolina, Charleston, SC 29425, USA; gry201@musc.edu (G.R.Y.);; 2 Department of Public Health Services, Medical University of South Carolina, Charleston, SC 29425, USA

**Keywords:** urology, oncology, women

## Abstract

**Background/Objectives**: The role of female surgeons in urology has been steadily increasing. We performed a contemporary review of American Board of Urology (ABU) case logs focused on oncologic procedures and evaluated the role of female surgeons over the past two decades. **Methods:** Operative logs from ABU examinees from 2003 to 2023 were analyzed. We identified open-approach (OA) and minimally invasive (MIS) radical nephrectomy (RN), partial nephrectomy (PN), radical nephroureterectomy (RNU), radical prostatectomy (RP), and adrenalectomy (RA) using CPT codes. Total case volumes as well as reported fellowship training were recorded and tabulated. The counts and proportions of OA and MIS procedures were analyzed over time and by surgeon gender. **Results**: From 2003 to 2023, 54,972 surgical procedures were reported to ABU with only 2.1% (1127) being performed by female surgeons. Of these, 32.5% (366) were OA and 67.5% (761) were MIS. Despite the low overall composition of female-performed procedures, the number of surgeries performed by females increased over time. Among female surgeons, the proportion of MIS surgeries increased over time, from 37.5% to 71.5% in 2003–2009 to 2017–2023, respectively. Females versus males performed comparably for OA for RN and RA; however, females performed more open PN, RNU, and RP than their male counterparts. Moreover, the number of procedures performed by oncology fellowship-trained females increased significantly. **Conclusions**: Our analysis of over twenty years of data submitted to the ABU indicates that the surgical volume of oncologic procedures by female urologists has been increasing. These findings demonstrate the increased contributions by female surgeons to the field urologic oncology.

## Simple Summary

This study examined trends in women’s involvement in urologic oncology over the past two decades using American Board of Urology case logs from 2003 to 2023. Among the nearly 55,000 oncologic surgeries performed, only 2.1% were performed by female surgeons; however, the number of cases performed by women annually has increased steadily. Female surgeons increasingly adopted minimally invasive surgery and also performed a higher proportion of open procedures than male surgeons for certain operations, including partial nephrectomy and radical prostatectomy. The number of oncology fellowship-trained female urologists rose throughout the twenty-year period. Overall, the findings support the growing contributions of women in urologic oncology and highlight the meaningful progress despite continued underrepresentation.

## 1. Introduction

The presence of female surgeons in urology has been steadily increasing, with one study showing an increase of 104% between 2007 and 2019 [1]. In 2023, 11.8% of practicing urologists were women, with 24.5% of urologists under 45 years of age being women [2]. Likewise, the number of women applying and joining the field of urologic oncology is growing annually [2].

A few studies in 2016 and 2017 assessed the practice patterns associated with surgeon gender and the procedures performed using the American Board of Urology (ABU) data, finding that female urologists performed significantly more female-specific procedures and male urologists performed significantly more male-specific procedures [3,4]. However, trends examining the relationship between surgeon gender and procedural approach specifically within urologic oncology have not previously been evaluated. As surgical practice continues to evolve, understanding whether surgeon gender is associated with differences in operative approach is important to highlight. This is particularly relevant in the context of growing efforts to promote gender equality and advocacy in the realm of urologic oncology with institutions like Women in Urologic Oncology (WUO) and the Society of Women in Urology (SWIU), which aim to diversify the urologic workforce and may amplify opportunities for gender concordant care and research. Herein, we performed a contemporary review of ABU case logs focused on oncologic procedures and evaluated the role of female surgeons over the past two decades.

## 2. Materials and Methods

Operative logs from ABU examinees from 2003 to 2023 for oncologic procedures performed on patients aged 18 years and older were analyzed. In total, 54,972 logs were obtained. We identified radical nephrectomy (RN), partial nephrectomy (PN), radical nephroureterectomy (RNU), radical prostatectomy (RP), and adrenalectomy (RA) using appropriate CPT codes and classified these into open-approach (OA) and minimally invasive (MIS). Additional information available for each case included surgeon gender, certification, fellowship, and log year as well as region of the procedure. Certification was dichotomized as ‘first certification’ (C1, F1, L1, M1, P1, ER) and ‘re-certification’ (C2, F2, L2, L3, M3, M4, P2, R1, R2, R3). Surgeon fellowship categories were collapsed into groupings based on fellowship type and accrediting body, which were agreed upon by the consensus of the study team based on clinical expertise. Table 1 provides a descriptive summary of all available case logs.

Log years were grouped into equally sized categories of 2003–2009, 2010–2016, and 2017–2023 to assess trends in the counts of surgeries and proportions of OA over time. Additionally, the proportions of surgeries that were OA were calculated within surgeon gender and compared across procedure type, with 95% Wald confidence intervals (CIs) constructed for the difference in proportions between male and female surgeons. Lastly, a complete case analysis (*n* = 54,959) was performed to estimate the probability of OA surgery using binary regression generalized linear model (GLM) with logit link and surgeon gender, certification, fellowship, log year, and region as independent variables. An odds ratio (OR) for gender was estimated to compare female and male surgeons’ use of OA. Predictor variables were chosen based on their prognostic value; the variance inflation factor was assessed for each predictor and no issues with collinearity or model fit were noted.

All calculations were performed in R version 4.4.1 with packages ggplot2 3.5.1 and cowplot 1.1.3 for plotting [5,6,7]. Differences in proportions for which the CI did not contain zero were deemed statistically significant and a *p*-value threshold of α ≤ 0.05 was used to assess the significance of the odds ratio from the GLM.

This study was Institutional Review Board exempt.

## 3. Results

**Figure 1:**
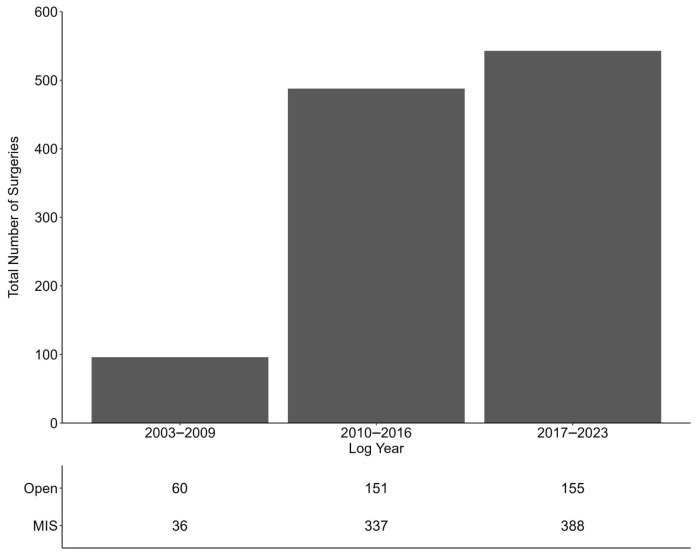
Total procedures performed by females over log year.

From 2003 to 2023, 54,972 oncological procedures were reported to ABU with only 2.1% (1127) being performed by female surgeons. Despite the low overall composition of female-performed procedures, the number of surgeries performed by females increased over time from 96 to 488 to 543 in year groups of 2003–2009, 2010–2016, and 2017–2023, respectively (Figure 1). Specifically, the number of cases rose from 0 in log year 2003 to 118 in 2022, with a peak in 2017 of 148 cases. Aggregating across all log years, the number of procedures performed by oncology fellowship-trained female surgeons rose from 37 cases in 2003–2009 to 101 in 2017–2023.

**Figure 2:**
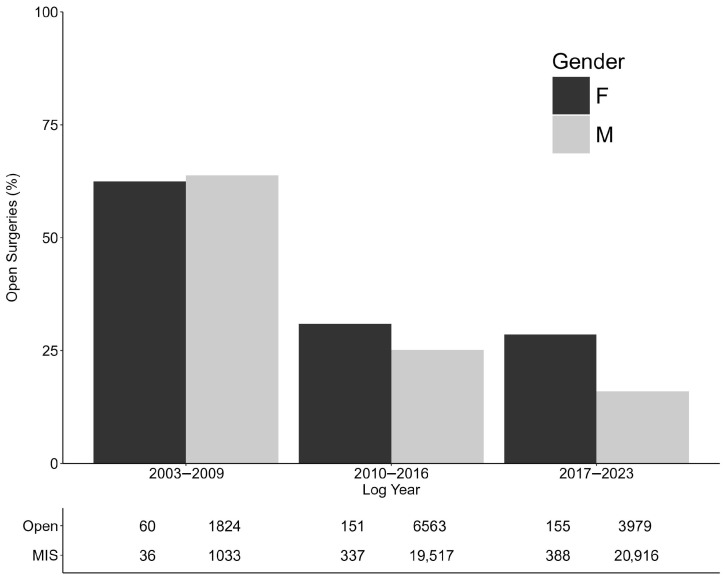
Percentage open surgeries by log year and gender.

Of the procedures performed by female surgeons, 32.5% (366) were open and 67.5% (761) were MIS (Figure 2). Across the same period, male surgeons reported proportionally less OA at 23% (12,366/53,832 oncologic procedures), female–male difference = 9.5%, 95% CI: 6.7% to 12.3% (Figure 2). Among female surgeons, the proportion of MIS surgeries increased over time, going from 37.5% to 69.1% to 71.5% in log year groups of 2003–2009, 2010–2016, and 2017–2023, respectively. The same increasing MIS trend was seen across the same time periods for RP. The other surgeries had fluctuations: RN (23.5% to 66.3% to 50.8%), PN (9.5% to 56.6% to 54.8%), RNU (50% to 49.4% to 65.5%), and RA (100% to 62.5% to 77.8%).

**Figure 3:**
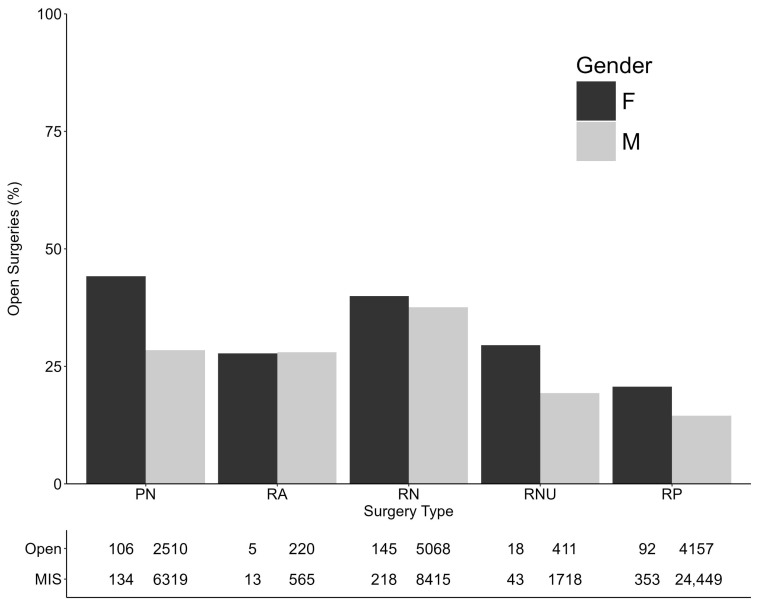
Open surgery by procedure type and gender.

Females performed similar proportions of OA compared to male surgeons across RN (39.9% [145/363] vs. 37.6% [5068/13,483], difference = 2.4%, 95% CI: −2.7% to 7.5%) and RA (27.8% [5/18] vs. 28.0% [220/785], difference = −0.2%, 95% CI: −21.2% to 20.7%) when compared to male surgeons (Figure 3). However, females performed proportionally more open PN (44.2% [106/240] vs. 28.4% [2510/8829], difference = 15.7%, 95% CI: 9.4% to 22.1%), RP (20.7% [92/445] vs. 14.5% [4157/28,606], difference = 6.1%, 95% CI: 2.3% to 9.9%), and RNU (29.5% [18/61] vs. 19.3% [411/2129], difference = 10.2%, 95% CI: −1.4% to 21.8%) than their male counterparts, although this difference was not statistically significant for RNU (Figure 3). On multivariable analysis, female surgeons had 1.65 times the odds of performing open procedures adjusted for log year, region, certification, and fellowship training (95% CI: 1.44 to 1.88; *p* < 0.01).

**Figure 4:**
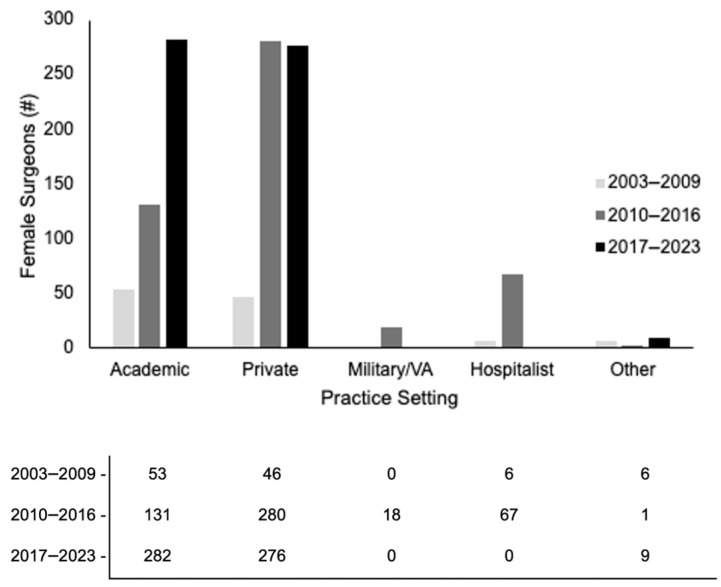
Total number of females in practice setting type.

**Figure 5:**
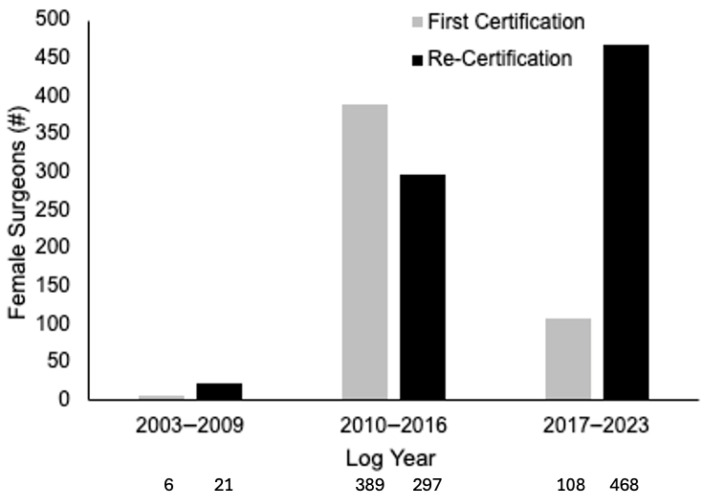
Total number of females per certification type.

The majority of women were employed in academics or private practice, with the number employed in 2017–2023 between the two being almost equal (Figure 4). Academic medicine had a steady rise between the study periods, whereas private practice had a sharp rise between the first two periods which stayed overall steady for the third period. The number of women that re-certified increased within each log group period (Figure 5).

## 4. Discussion

According to the 2023 Center for Medicare and Medicaid Services National Downloadable Database, there is a total of 1,168,064 physicians with 7.3% being surgeons and the remaining 92.7% comprising medicine specialties [8]. Boutros et al. further report that women outnumber men 51.3% to 48.7% when looking at physicians as a whole [8]. However, the same study highlights that separating surgeons from medicine doctors shows a stark contrast. Only 16.7% of surgeons identify as female in contrast to 54% of non-surgeons identifying as female [8]. Moreover, only 12.6% (1184) of urologists are female and, in comparison to general surgery, there is a significantly lower likelihood of females choosing urology [8]. However, between 2007 and 2019 there was an increase by 104% in female urology trainees and the number of women matriculating into medical school is now higher than 50% [1,9].

Ketley and Morgan performed a review as they questioned why females outnumber male medical students, but the number of women in surgical specialties continues to fall behind non-surgical specialties [10]. They specifically assessed oncologic surgery specialties as they noted that cancer surgery has conventionally been predominantly male. They noted that female surgeons are faced with unique challenges during their career; for example, surgical trainees report that returning to work after maternity leave causes several challenges including poor assumptions about loss of skills and perceived commitment level [10]. Potentially, these factors may impact the surgical volume or operative approach such as the ability to build and sustain high-volume practices.

Linscheid et al. assessed over four decades of data from the Association of American Medical Colleges (AAMC) and found that, compared with general surgery, more specialized fields of surgery showed a slower growth in the percentage of women comprising that specialty specifically for neurosurgery, orthopedic surgery, cardiothoracic surgery, and urology [11]. This was converse to specialties with an increasing percentage of females including obstetrics and gynecology, ophthalmology, and otolaryngology [11]. Expanding on this, Jackson et al. created a two-parameter logistic growth model and found that the percentage of female graduates from medical school matriculating into urology residency is rising by 0.6% annually [12]. Given the rising number of women in urology, Jackson et al. report that it is estimated that by the year 2062, 38% of practicing urologists will be female [12]. Similarly, in the Canadian system, Pickel and Sivachandran assessed MD applicants, students, graduates, residents, and practicing surgeons, finding that though there are more female graduates than males, there are significantly fewer practicing surgeons [13]. Specific to urology, the rate of increase in female representation was 0.37% in contrast to obstetrics and gynecology at 1.59%, general surgery at 0.88%, plastic surgery at 0.71%, and otolaryngology at 0.64% annual rise [13]. Fields similar to urology were ophthalmology, orthopedic surgery, neurosurgery, and cardiac surgery with yearly increase rates of 0.48, 0.36, 0.36, and 0.18%, respectively [13]. Similarly to Jackson’s group, Pickel et al. created a logistic growth model, showing that with the current rates, it will be a century before gender equality is seen in urology, neurosurgery, and orthopedics, with two centuries predicted for cardiac surgery [13].

Interestingly, Boutros et al. found that women were most represented in surgical oncology, with 44.3% of surgeons identifying as female [8]. On the opposite end of the spectrum, Tsilimigras and colleagues assessed nearly 12,000 oncologic surgery Medicare claims and found that only 17.7% of the surgeons were female [14]. Concerningly, the proportion of cases managed by female surgeons decreased when going from early to late career, whereas the proportion remained relatively stable for male surgeons [14].

The trends in the papers above show a gradual but meaningful transformation in the field of urologic oncology with respect to female surgeons. While females remain under-represented at only 2.1%, there is a clear and sustained progress in the number of female urologists and an increase in oncologic procedures performed by female surgeons.

Collectively, we found that though MIS are increasing among urologic oncology procedures, differences in operative approach by surgeon gender do exist. Our study using ABU case logs shows that the surgical volume of urologic oncological procedures performed by female surgeons has increased over the past two decades. Despite female surgeons performing only 2.1% of urologic oncology procedures, the number of procedures has been annually increasing and will continue to increase as more women continue to become urologists. The higher likelihood of OA remained significant even after adjusting for training, certification status, and geography. This is important as several studies, including a large meta-analysis by Saka et al., found a lower mortality trend for patients treated by female surgeons regardless of the nature of the case (elective versus non-elective) [15].

Our study found that female surgeons had a higher likelihood of performing OA in comparison to males. This is important as open surgery is becoming increasingly rare across different procedures and is being replaced by minimally invasive approaches. Open surgery skills remain paramount for managing complex oncologic cases, but the exposure to open techniques continues to decline during residency training. Several studies in general surgery have reviewed Accreditation Council for Graduate Medical Education (ACGME) National Operative Case Logs and compared open versus minimally invasive surgeries (MIS), finding that while the total average case volumes have increased significantly, MIS account for a larger percentage of these procedures [16,17]. Urology has been shown to be the specialty with the highest proportion of robotic surgery use, comprising 34.1% in a 2018 paper using the Nationwide Inpatient Sample Database [18].

This is the first study that assessed the effect of surgeon gender on surgical approach for all major urologic oncology procedures with existing CPT codes, and we found that females performed more open PN and RNU than their male colleagues. Female surgeons performing a higher proportion of open procedures may reflect case selection, patient factors, and practice settings, rather than a decreased adoption of MIS. In 2023, Passarelli et al. reported their findings comparing Centers for Medicare and Medicaid Services (CMS) data on open versus robotic/laparoscopic surgery for multiple specialties [19]. Within urology, they assessed the approaches of RP, RN, PN, and sacrocolpopexy (SCP). Their study found that the highest number of women performing MIS was in gynecology at 39.5% with the lowest involvement in urology at 4.05% [20]. Moreover, when assessing oncologic procedures including RP, PN, and RN MIS approaches, the percentages of women performing these procedures were all < 2% [19]. For SCP, women performed a higher amount of MIS at 31.9% [19]. Overall, for the surgeries that were studied, women performed 3.5% of MIS [19]. A similar study by Passarelli et al. assessed SBU case logs between 2012 and 2022, finding that women performed 7.2% of MIS surgeries [20]. This was further stratified into female urologists performing 36.9% and pediatric urologists performing 23.8% [20]. Oncology had the lowest number of women performing MIS at 3.4% [20].

Our study shows that the number of procedures performed by oncology fellowship-trained females is increasing. This is in contrast to a prior study by Netty et al. in 2018 who performed a review of ABU case logs between 2004 and 2015 to assess surgeon gender and practice characteristics [21]. During this time period, only 34 women versus 603 men identified themselves as oncology sub-specialists, with males being 1.7 times more likely to report an oncologic sub-specialty over females [21]. Conversely, andrology and infertility had equal sub-specialty representation, whereas pediatric and female urology had significantly more women subspecializing than their male counterparts [21]. Our study supports that the number of cases being performed by female surgeons is increasing, and moreover that the number of procedures performed by oncology-trained female surgeons is increasing. The greater representation of female oncology-trained urologists is encouraging and because being given mentorship is a predictor in female medical students choosing urology, this can improve recruitment and inclusive training environments.

The strength of this study is that it is representative of urologists requesting board certification or re-certification over the twenty-year period. This study is not without limitations. Methodologically, the analyses focused on descriptives breaking down rates of open surgery across gender and within surgeon factors. Although the regression model provides a high-level comparison of gender adjusted for additional surgeon factors, a more robust modeling approach assessing the interactions between gender and the remaining predictor variables over time would provide key insights into aspects of the data that the descriptive proportions miss.

The data are self-reported, may contain incomplete records, and female surgeons, the key group of interest, only comprise a small proportion of the sample. Female and male comparisons within levels of surgeon factors often resulted in small sample sizes, leading to wide confidence intervals. Statistical significance when noted should be taken in mind with the estimated difference and its confidence interval. Notably, open RA procedures for females had less than ten cases. ABU case logs only capture procedures submitted for certification and therefore may not fully reflect a surgeon’s complete operative volume. Given this, there could be a bias that differs by gender when logging cases for certification.

Moreover, the case logs do not include patients’ medical past or surgical history, which can guide surgeons to perform one approach over another. Likewise, the case logs do not report disease-specific data such as tumor size, stage, location, or complexity. Additionally, given the nature of the self-reporting system, it is unable to assess the postoperative outcomes on patients treated by female versus male urologists. Interestingly, several studies have assessed postoperative outcomes, finding that female surgeons have improved outcomes for mortality, readmission, and complications compared to their male colleagues [16,22]. With these limitations in mind, the ABU data remain one of the most comprehensive ways to assess the practice patterns of urologists in the United States.

This study addresses the surgeon genders as binary due to the nature of reporting in the ABU database, which reflects historical limitations in demographic data collection. In 2021, it was estimated that ~1.2% of medical students identified as gender expansive, but there is a lack of data as most studies do not include those outside of the gender binary [23]. Within urology, a survey was sent out to New York medical schools, showing that 15% were underrepresented minorities (URMs), 19% identified as a member of the LGBTQIA+ community, and 3.5% identified as both [24]. Both URM and LGBTQIA+ students reported the lack of diversity within urology and the exclusivity of urology as being barriers in comparison to other students [24].

The first year that the American Urologic Association started collecting gender demographics outside of the binary genders was 2021. In 2021, 48 individuals out of over 13,000 urologists identified as nonbinary, transgender, or other gender identity [25]. As more nonbinary and transgender people have been matriculating into medical school, these numbers would be expected to rise in urology as well [26]. As the recognition of gender diversity improves within medicine and surgery, improved data collection and reporting will be important to better understand practice patterns.

## 5. Conclusions

Ultimately, our analysis of the last two decades of data submitted to the ABU indicates that the surgical volume of oncologic procedures by female urologists has been increasing. As more women enter urology and further pursue urologic oncology, their contributions will continue to shape the evolving field. Moreover, women continue to integrate both open and minimally invasive approaches into the changing surgical landscape. These findings underscore not only where the specialty stands today, but also where opportunity lies again. With this in mind, the future of women in urologic oncology is indeed bright.

## Figures and Tables

**Table 1:** Characteristics of case logs.

Surgeon Gender	
F	1127 (2.1%)
M	53,832 (97.9%)
Unknown	13 (<0.1%)
**Certification**	
First Certification	15,641 (28.5%)
Re-Certification	39,331 (71.5%)
**Fellowship**	
Endourology/Laparoscopy/MIS	4832 (8.8%)
Oncology	5957 (10.8%)
Reconstructive/Female	829 (1.5%)
Other	1028 (1.9%)
None	42,326 (77.0%)
**Log Year**	
2003–2009	2953 (5.4%)
2010–2016	26,568 (48.3%)
2017–2023	25,451 (46.3%)
**Region**	
International	243 (0.4%)
Mid-Atlantic	5572 (10.1%)
North Central	12,111 (22.0%)
New England	7711 (14.0%)
South Central	8301 (15.1%)
Southeastern	11,615 (21.1%)
Western	9419 (17.1%)
**Surgery Type**	
Adrenalectomy	804 (1.5%)
Nephroureterectomy	2190 (4.0%)
Partial Nephrectomy	9073 (16.5%)
Radical Nephrectomy	13,850 (25.2%)
Radical Prostatectomy	29,055 (52.9%)

## Data Availability

The data were obtained from the American Board of Urology. This is not a publicly archived dataset and must be obtained directly from the American Board of Urology with a research proposal.

## Abbreviations

The following abbreviations are used in this manuscript:

ABU	American Board of Urology
OA	Open Approach
MIS	Minimally Invasive
RN	Radical Nephrectomy
PN	Partial Nephrectomy
RNU	Radical Nephroureterectomy
RP	Radical Prostatectomy
RA	Radical Adrenalectomy
